# Characterization of calcium sources and downstream signaling pathways following electrical field stimulation of rodent longitudinal esophageal muscle

**DOI:** 10.3389/fphys.2026.1907835

**Published:** 2026-08-19

**Authors:** Michelle Gräfe, Julius-Neven Kriese, Timo Kirschstein, Rüdiger Köhling, Felix Bock, Felix Schulze, Guido Hildebrandt, Bernd Frerker

**Affiliations:** 1 Oscar Langendorff Institute of Physiology, University Medicine Rostock, Rostock, Germany; 2 Center of Transdisciplinary Neurosciences Rostock (CTNR), University Medicine Rostock, Rostock, Germany; 3 Department of Radiation Oncology, Rostock University Medical Center, Rostock, Germany; 4 Department of Pediatric Surgery, Rostock University Medical Center, Rostock, Germany

**Keywords:** calmodulin, chelerythrine, electrical field stimulation, IP3 receptor, protein kinase C, rodent esophageal muscle, verapamil, W-7

## Abstract

**Aims:**

To explore the mechanisms involved in esophageal body longitudinal muscle contraction induced by electrical field stimulation.

**Methods:**

Isometric contractions of esophageal segments from wistar rats in an organ bath were induced by electrical field stimulation (duration 1 s, frequency 1-100 Hz, intensity 30-90 V) before and after application of pharmacological probes to test involvement of muscarinic receptors, Rho kinase, Ca^2+^ release, protein kinase C, calmodulin and L-type Ca^2+^ channels.

**Results:**

Electrical field stimulation (EFS) induced contractions showed a frequency and intensity-dependent behavior. Based on the effect size, expressed by the Cohen’s d, they were insensitive to the muscarinic receptor blocker atropine (1 µM), the Rho kinase inhibitor Y-27632 (10 µM) as well as the inhibitors of Ca^2+^ release 2-aminoethoxydiphenylborane (2-APB, 100µM) and 1,1’-diheptyl-4,4’-bipyridinium (DHBP, 100 µM). In contrast, contractions were reduced by the protein kinase C inhibitor chelerythrine (10µM) and by the calmodulin antagonist N-[6-aminohexyl]-5-chloro-1-naphthalenesulfonamide hydrochloride (W-7, 100 µM). Verapamil (100 µM) abolished EFS-induced contractions.

**Conclusions:**

Based on our findings with 2-APB, DHBP, and verapamil, extracellular Ca²^+^ appears to be the source of the increase in intracellular Ca²^+^ underlying EFS-induced EB contractions. Furthermore, the effects observed with chelerythrine and W-7 may suggest that this increase in intracellular Ca²^+^ may subsequently activate PKC- and calmodulin-dependent signaling pathways.

## 1 Introduction

Physiological contractions of the esophagus include peristalsis by esophageal body (EB) motility and control of esophagogastric transport by lower esophageal sphincter (LES) tone (Goyal and Chaudhury, 2008). Up to now, the latter has attracted most attention given its clinical relevance in gastroesophageal reflux disorder, and research unraveled signaling molecules including nitric oxide (Kuramoto et al., 2013; Shimbo et al., 2016), hydrogen sulfide (Bai et al., 2018), vasoactive peptides like angiotensin II (Rattan et al., 2003), and others (Saegusa et al., 2011; Ghasemi-Kasman et al., 2012; Shiina et al., 2016; Bai et al., 2019; Morishita et al., 2023). In contrast, understanding of EB motility mechanisms is largely limited to vagal nerve involvement, based on studies showing that acetylcholine or carbachol (parasympathomimetic that acts as a chemically stable acetylcholine analog) contracts circular and longitudinal muscle strips across species, including rats (Soyer et al., 2010; Hançerlioğullari et al., 2012; Soyer et al., 2015; Frerker et al., 2022; Shiina et al., 2024), cats (Park et al., 2009c), other animals (Ackbar et al., 2012; Igarashi-Hisayoshi et al., 2023), or humans (Tøttrup et al., 1990b; González et al., 2000). In this context, muscarinic receptors, protein kinase C, Rho kinase, calmodulin, and calcium have been implicated in esophageal smooth muscle contraction (Murthy, 2006; Harnett et al., 2005; Harnett and Biancani, 2003; Sohn et al., 2003, Sohn et al, 2001; Cao et al., 2001; Kim et al., 2004). Moreover, the source of the calcium required for gastrointestinal smooth muscle contraction in animals (Harnett and Biancani, 2003; Sohn et al., 2003, Sohn et al., 2001; Cao et al., 2001; Sohn et al., 2000, Sohn et al., 1994) and humans (Hurley et al., 1999; Sims et al., 1997) is well characterized.

Besides agonist application, electrical field stimulation (EFS) is an additional tool to induce esophageal muscle contraction. Similar to pharmacological stimulation, EFS induces contractions of the LES or EB across species, including rats (Ghasemi-Kasman et al., 2012; Akbarali et al., 1988a; Storr et al., 2001; Nakamori et al., 2012), cats (Park et al., 2009c, Park et al., 2010, Park et al., 2009a, Park et al., 2009b), rabbits (Koc et al., 2024; Capanoglu et al., 2016), other animals (Shiina et al., 2016, Shiina et al., 2015, Shiina et al., 2012; Lecea et al., 2012; Boudaka et al., 2007; Muinuddin and Paterson, 2001) and humans (Lecea et al., 2011; González et al., 2004; Tøttrup et al., 1990a), sharing features with cholinergic agonist–induced contractions in airways (Wigenstam et al., 2021) or bladder (Stenqvist et al., 2020). However, EFS-induced contractions can also be elicited in smooth muscle tissues lacking cholinergic contraction, such as arteries (Darios et al., 2016), indicating tissue specificity and suggesting distinct pathways for EFS- and cholinergic agonist–induced contractions. Following EFS, atropine, a muscarinic receptor antagonist, shows region- and (smooth or striated) muscle-dependent effects: while several studies report atropine sensitivity (Park et al., 2009c; Storr et al., 2001; Nakamori et al., 2012; Park et al., 2010, Park et al., 2009a, Park et al., 2009b; Koc et al., 2024; Shiina et al., 2015; Lecea et al., 2012; González et al., 2004; Tøttrup et al., 1990a), some others did not (Ghasemi-Kasman et al., 2012; Storr et al., 2001; Nakamori et al., 2012; Shiina et al., 2015, Shiina et al., 2012; Boudaka et al., 2007; Muinuddin and Paterson, 2001; Zhang et al., 2018).

Data on further mechanisms of EFS-induced contractions are scarce and mainly derive from Park et al., who distinguished off- and on-contractions: off-contractions occurred after EFS termination, whereas on-contractions occurred after EFS onset in the presence of a nitric oxide synthase (NOS) inhibitor (Park et al., 2009a). The protein kinase C (PKC) inhibitor chelerythrine did not affect off-contractions in the distal feline smooth esophagus (Park et al., 2009b), but inhibited on-contractions when NOS was blocked (Park et al., 2010). Furthermore, the Rho kinase (ROCK) inhibitor Y-27632 and the myosin light chain kinase (MLCK) inhibitor ML-9 inhibited both contractions (Park et al., 2009c, Park et al., 2010, Park et al., 2009b), but another study found no effect (Capanoglu et al., 2016).

Although EFS-activated cholinergic contraction may involve Rho kinase, MLCK or PKC in esophageal smooth muscle, there are only limited data available on the striated muscle of rats. To the best of our knowledge, there are only a few older studies on the effect of calcium on esophageal contraction in rats (Akbarali et al., 1988a, Akbarali et al., 1988b); however, these studies merely addressed only the smooth muscle of the tunica muscularis. Therefore, we used different compounds to investigate EFS-induced contractions in rat EB longitudinal striated muscle, and we aimed to identify the required calcium source.

## 2 Methods

### 2.1 Rat esophagus preparation

In total, 27 adult male Wistar rats (7.6 ± 2.2 months old, range 2−11 months, Charles-River, Sulzfeld, Germany) were used for this study. After deep anesthesia with diethylether, rats were decapitated. The abdomen was incised to locate the gastric cardia and identify the abdominal esophagus, followed by opening the thorax to identify thoracic and cervical segments; the entire esophagus was excised and submerged in 4°C dissection solution consisting of (in mM): 145 NaCl, 4.5 KCl, 1.2 NaH_2_PO_4_, 0.1 CaCl_2_, 1.0 MgSO_4_, 0.025 Na_2_-EDTA, and 5 HEPES (pH=7.4). In this solution, the esophagus was cut into 1 cm specimens (proximal, middle, distal), yielding up to six specimens per rat. The specimens (in total n=156) were provided with nylon threads to fix them in an organ bath at 37°C (volume 25 ml) filled with the following organ bath solution (in mM): 120 NaCl, 4.7 KCl, 2.5 CaCl_2_, 1.2 MgCl_2_, 30 NaHCO_3_, 0.5 Na_2_-EDTA, 5.5 glucose, 2 sodium pyruvate. This solution was gassed with carbogen (95% O_2_ and 5% CO_2_) to maintain the pH at 7.4. The whole procedure from decapitation to insertion in the organ bath chamber lasted less than 30 min.

### 2.2 Organ bath measurement

After insertion into the organ bath, esophageal specimens were left untensioned to adapt to buoyancy and solution conditions. Within 30 min of adaption, specimens were gently stretched to 2–3 mN to ensure transducer sensitivity for isometric contractions and relaxations, as moderate preload facilitates EFS-induced contraction (Soyer et al., 2015). After adaptation, the esophageal specimens were allowed to produce a spontaneous basal tone which was determined after 30 min of equilibration (i.e. 60 min after organ bath insertion). The organ bath (Panlab ML0146/C), the force transducers (Panlab MLT0201), the bridge amplifiers (Panlab ML224), the analogue-to-digital converter (PowerLab 4/30), and the software (Labchart, version 8, RRID: SCR_023643) were purchased from AD Instruments (ADI, Spechbach, Germany, RRID: SCR_001620).

With our organ baths, each equipped with four chambers for measuring isometric force, the volume of each chamber is 25 ml. To test any substance effect on the organ, the compound has to be manually applied using a pipette. Diffusion into the organ is facilitated by continuous bubbling with carbogen. To remove any compound from the organ bath, one has to refresh the organ bath solution by opening a valve and letting the solution drain out. Both drain and refill are driven by gravity. Thus, it takes roughly 2–5 s for the chamber to be drained and the same amount of time to refill it with fresh solution.

### 2.3 Electrical field stimulation

To analyze the effects of various compounds on EFS-induced contraction, we always used the following protocol: At the beginning of the experiment (i.e. 60 min after organ bath insertion), the specimens showed an average baseline tone of 2.85 ± 0.03 mN (n=156). Despite randomization regarding the compound assigned for subsequent testing, some variability was observed among the experimental groups (**Supplementary Figure 1A**). First, the specimens were exposed to 60 mM KCl (termed KCl Start) for 10 min by adding 1000 µl of 1.5 M stock solution to the organ bath (volume 25 ml) which resulted in a robust contraction (peak 7.3 ± 0.3 mN, n=156). Posthoc comparison of the three segments (proximal, middle, distal) revealed no statistically significant differences (regarding c_max_ reduction, see **Supplementary Table 1**). After washout for 25 min, we delivered an electrical field stimulation (EFS) paradigm using eight trains of 1 s duration with increasing frequencies (1 Hz to 100 Hz, rectangular pulses of 100 µs duration) separated by 20 s. All trains were repeated with increasing voltages (10−90 V in 10-V-intervals, 40 s apart). Since EFS with simulation below 30 V never produced any contractions, we decided to use the voltage range 30−90 V (after 18 from 27 animals). EFS was produced by a stimulus isolator (ISO-STIM-01D, npi electronic, Tamm, Germany) via a stainless steel stimulation electrode (PanLab LE0130A). The EFS-induced contractions differed slightly between proximal, middle and distal segments (**Supplementary Figure 2**). Although these differences reached statistical significance (three-way ANOVA with Holm-Sidak test), the effect sizes were rather small (proximal versus middle: 0.37, proximal versus distal: 0.20, middle versus distal: 0.17). Hence, we decided that these differences should not be regarded as meaningful. Following the EFS paradigm (duration 20−25 min), the organ bath solution was renewed and one of the pharmacological probes (see chapter 2.4) or vehicle (distilled water or DMSO) was applied and allowed to incubate for at least 10 min. Then, the entire EFS paradigm (i.e. starting 130−140 min after insertion in organ bath) was repeated. All EFS-induced contractions were normalized to the initial KCl response. Finally, all specimens were again challenged with 60 mM KCl (starting 3 hours after organ bath insertion), which gave rise to a final KCl response (termed KCl End) of 4.0 ± 0.2 mN (64 ± 4% of KCl Start, n=156). A detailed comparison of the KCl End contraction relative to the KCl Start contraction is shown in **Supplementary Figure 1B**.

### 2.4 Chemicals

Chemicals used for both the dissection and the organ bath solution including KCl were obtained from Sigma-Aldrich. All other chemicals were purchased from Tocris (Bio-Techne, Wiesbaden, Germany). To study the mechanisms of EFS-induced contraction, the following test compounds were (pharmacological probes) used: the muscarine receptor blocker atropine (1 µM (Kirschstein et al., 2014; Mader et al., 2016; Park et al., 2009a; Storr et al., 2000, Storr et al., 2001)), the Rho kinase inhibitor Y-27632 (10 µM (Kirschstein et al., 2014; Park et al., 2009c; Capanoglu et al., 2016)), the inositol trisphosphate receptor blocker 2-aminoethoxydiphenylborane (2-APB, 100 µM (Kirschstein et al., 2009)), the ryanodine receptor blocker 1,1’-diheptyl-4,4’-bipyridinium (DHBP, 100 µM (Kirschstein et al., 2009)), the calmodulin antagonist N-[6-aminohexyl]-5-chloro-1-naphthalenesulfonamide hydrochloride (W-7, 100 µM (Strittmatter et al., 2011; Mustafa et al., 1999)), the protein kinase C inhibitor chelerythrine (10 µM (Park et al., 2009b, Park et al., 2010)), and the L-type Ca^2+^ channel blocker verapamil (100 µM (Mader et al., 2016; Ortiz et al., 1988; Huang et al., 2011; Burchés et al., 1992)). For all test compounds, we prepared stock solutions in order to add 100 µl to the organ bath volume of 25 ml (i.e. dilution 1:250). Except for 2-APB, DHBP and chelerythrine, which were dissolved in DMSO, distilled water was used to prepare stock solutions. Accordingly, control experiments were conducted using distilled water (time control) or DMSO (control). Pre-Incubation time was 10 min for all tested compounds as described before (Harnett et al., 2005; Sohn et al., 2003; Smid and Blackshaw, 2000; Oh et al., 2014; Kirschstein et al., 2014, Kirschstein et al., 2009; Mader et al., 2016). In **Supplementary Figure 3** (unpublished own data) we demonstrate that pre-incubation with Y-27632 for 10 min is sufficient to reduce the maximal contraction strength. Furthermore, in a recent study (Frerker et al., 2022), we demonstrated an immediate effect of carbachol-induced contraction of the rat esophagus, and the longest peak latency (time interval between adding CCH and the maximal contraction strength) was approximately 6 minutes. Thus, we decided to use a 10 min incubation time.

### 2.5 Statistics

Raw data were KCl-induced and EFS-induced contractions recorded with LabChart software. In addition, we normalized EFS-induced contractions to the first KCl response (KCl Start). Then, all absolute or KCl-normalized values of one animal with identical stimulation were aggregated to one value. All statistical analyses were thus performed in an animal-based fashion (n=number of segments, N=number of animals). Throughout the manuscript, all values are expressed as mean values ± standard error of the mean (SEM). In all figures, frequency-contraction curves show data for 30 V, 50 V, 70 V, and 90 V only. For all statistical analyses, however, data from 30 V, 40 V, 50 V, 60 V, 70 V, 80 V and 90 V were included. They included (1) three-way analysis of variance (three-way-ANOVA), (2) the maximum contraction, and (3) the effect size (Cohen’s d). While the first analysis (three-way ANOVA) was performed on KCl Start-normalized EFS-contractions, the other two (maximum contraction and effect size) were performed on absolute values.

First, three-way ANOVA with Holm-Sidak post-hoc test (SigmaStat 3.5 software) was performed on the full stimulation range (30−90 V, 1−100 Hz) using the factors treatment (i.e. absence or presence of the test compound), voltage and frequency. However, given P values refer to the factor treatment only, as significant effects by voltage or frequency were regarded as no meaningful.

Second, the maximum contraction (termed “*C_max_*”) was calculated as the arithmetic mean of the contractions induced by 80 Hz and 100 Hz (for the voltage range 60−90 V): Hence, we obtained four values of *C_max_*, for 60 V, 70 V, 80 V and 90 V, respectively.

To determine the effect on *C_max_* for different segments of the esophagus, we defined the relative.


$$C_{max}\;reduction=1-\frac{C_{max,probe}}{C_{max,baseline}}$$

which was calculated for 60−90 V. These four values were then averaged to result in the mean *C_max_reduction*.

Third, the effect size, Cohen’s d, was separately calculated for 60 V, 70 V, 80 V and 90 V as.


$$d=\frac{C_{max,1}-C_{max,2}}{\sqrt{\frac{(n_{1}-1)\times Var_{1}+(n_{2}-1)\times Var_{2}}{\left(n_{1}+n_{2}-2\right)}}}$$

where *C_max_*, *n* and *Var* denote the maximum contraction, the number and the variance of both groups (i.e. in most cases probe and baseline), respectively. The mean effect size of a given probe was then calculated as the arithmetic mean of the four effect size values (60−90 V).

### 2.6 Additional software

All figures were created using CorelDRAW Graphics Suite 2026 (version 27.0.0.121, RRID: SCR_014235). The static analysis was performed using SigmaStat (version 3.5, RRID: SCR_010285).

## 3 Results

### 3.1 EFS induces atropine- and Rho kinase-insensitive contractions

**Figure 1:**
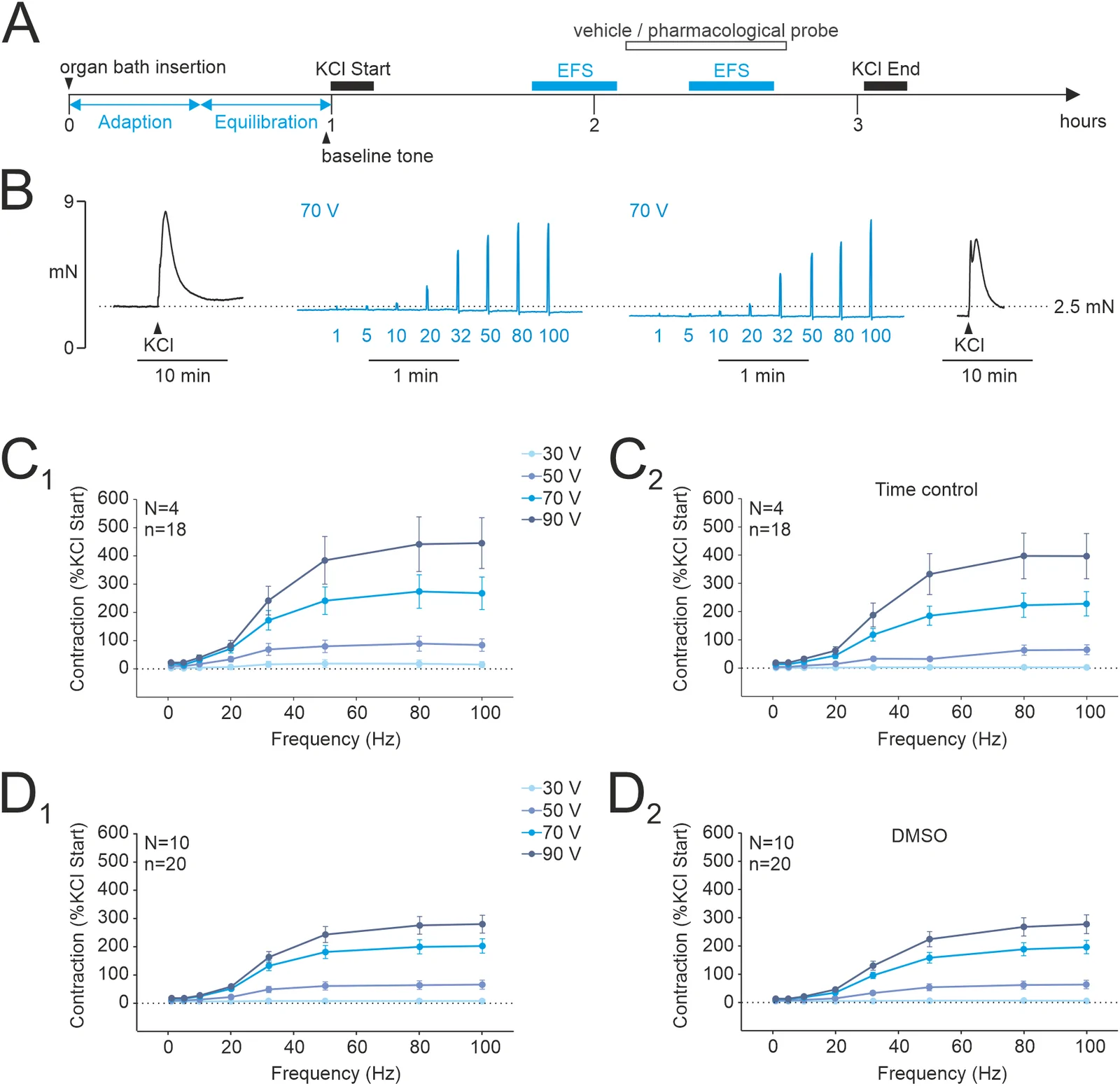
Electrical field stimulation (EFS) induces voltage- and frequency-dependent contractions. **(A)** Experimental timeline showing the equilibration period, the time point for determining the baseline tone as well as the applications of KCl, EFS and vehicle. Note that vehicle (here distilled water or DMSO) was replaced by various test pharmacological probe compounds in the following figures. **(B)** Representative force recording consisting of 60 mM KCl (KCl Start), twice EFS (first baseline, then under vehicle conditions), and again 60 mM KCl (KCl End). **(C)** Frequency-response curves for different voltages (30, 50, 70 and 90 V) either before **(C_1_)** or after distilled water (i.e. time control) as vehicle **(C_2_)**. **(D)** Frequency-response curves for different voltages (30, 50, 70 and 90 V) either before **(D_1_)** or after DMSO as vehicle **(D_2_)**. Blue numbers indicate the frequency that was used. N refers to the number of animals, while n denotes the number of esophageal segments used in the experiments.

We first applied 60 mM KCl to induce a depolarization-induced contraction for normalization purposes (Kirschstein et al., 2009) (“KCl Start” in **Figure 1A**). Then, EFS was delivered and produced contractions that depended on both stimulation frequency and voltage. An example using stimulation with 70 V at different frequencies is depicted in **Figure 1B**. To determine the effect of various test compounds, we applied either vehicle or pharmacological probes and repeated the EFS protocol (**Figure 1A**). Our control experiments with vehicle (distilled water, time control, N=4, n=18); and DMSO, control, N=10, n=20) during the second EFS protocol showed a small reduction compared to the first EFS protocol when normalized to the individual KCl Start response (**Figures 1C, D**). Albeit both treatment effects were statistically significant in the pre-treatment versus post-treatment comparison (P<0.01, 3-way ANOVA with Holm-Sidak test), the mean effect sizes were rather small (0.56 and 0.13 for distilled water and DMSO, respectively).

**Figure 2:**
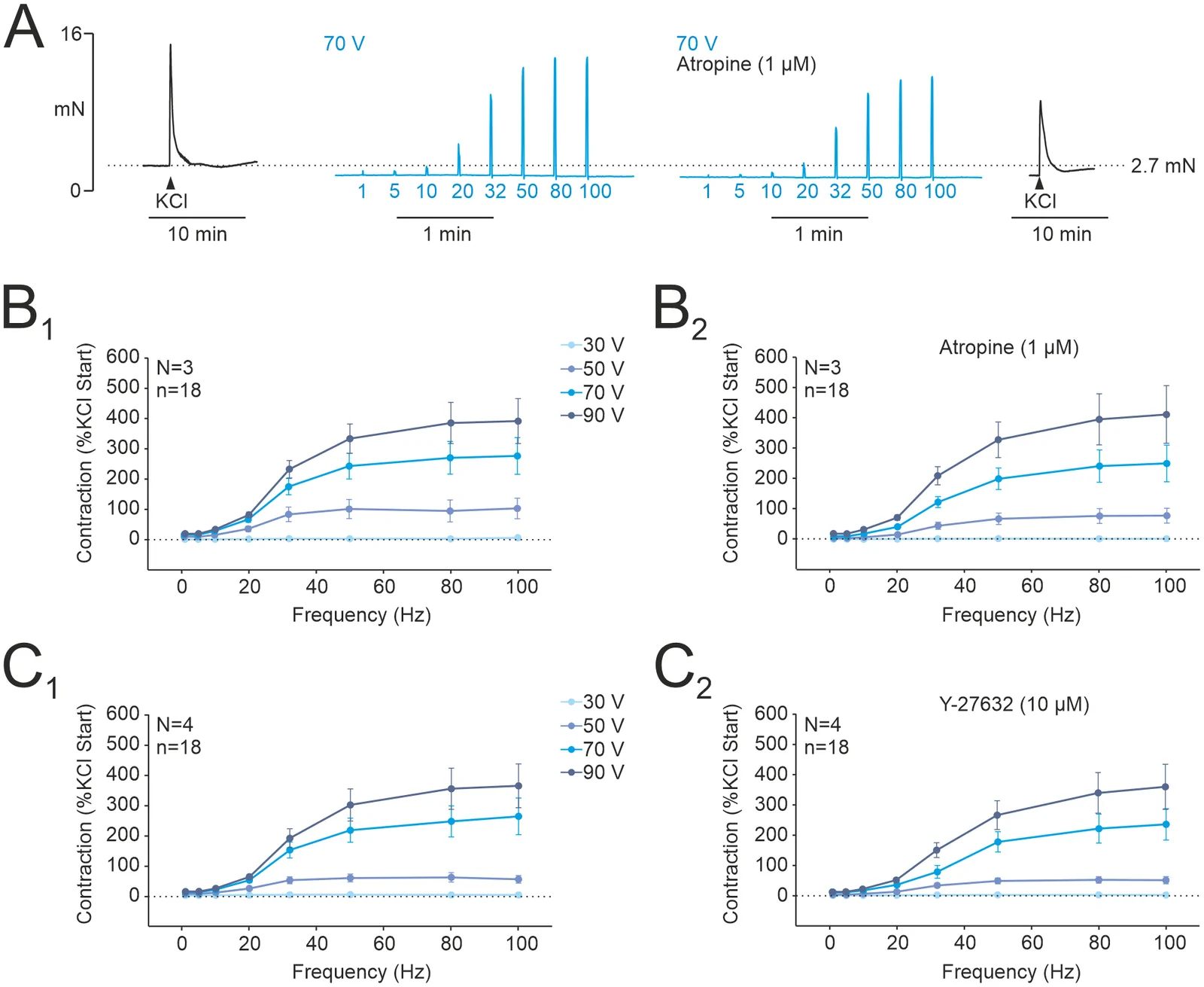
EFS-induced contractions are insensitive to atropine and Y-27632. **(A)** Representative force recording consisting of 60 mM KCl, twice EFS (first baseline, then under atropine), and again 60 mM KCl. **(B)** Frequency-response curves for different voltages (30, 50, 70 and 90 V) either before **(B_1_)** or after atropine **(B_2_)**. **(C)** Frequency-response curves for different voltages (30, 50, 70 and 90 V) either before **(C_1_)** or after Y-27632 **(C_2_)**. Blue numbers indicate the frequency that was used. N refers to the number of animals, while n denotes the number of esophageal segments used in the experiments.

To examine mechanisms of EFS-induced contraction, we first tested involvement of vagal nerve fibers in esophageal longitudinal muscle. Since muscarinic receptors may couple to Rho kinase (Sohn et al., 2000; Anderson et al., 2014), we applied the muscarinic receptor antagonist atropine (1 µM, N=3, n=18, **Figures 2A, B**) or the Rho kinase inhibitor Y-27632 (10 µM, N=4, n=18, **Figure 2C**). An example using stimulation with 70 V at different frequencies before and after adding atropine is depicted in **Figure 2A**. As shown in **Figures 2B, C**, both had minimal effects on EFS-induced contractions; although statistically significant in the case of atropine, when comparing pre- and post-treatment EFS curves (atropine: P<0.001, Y-27632: P=0.079, 3-way ANOVA with Holm–Sidak test), mean effect sizes were small (0.14 for atropine, 0.36 for Y-27632). Thus, muscarinic receptor and downstream Rho kinase activation cannot be fully excluded but are unlikely to play a major role.

### 3.2 Investigation of internal Ca^2+^ release

**Figure 3:**
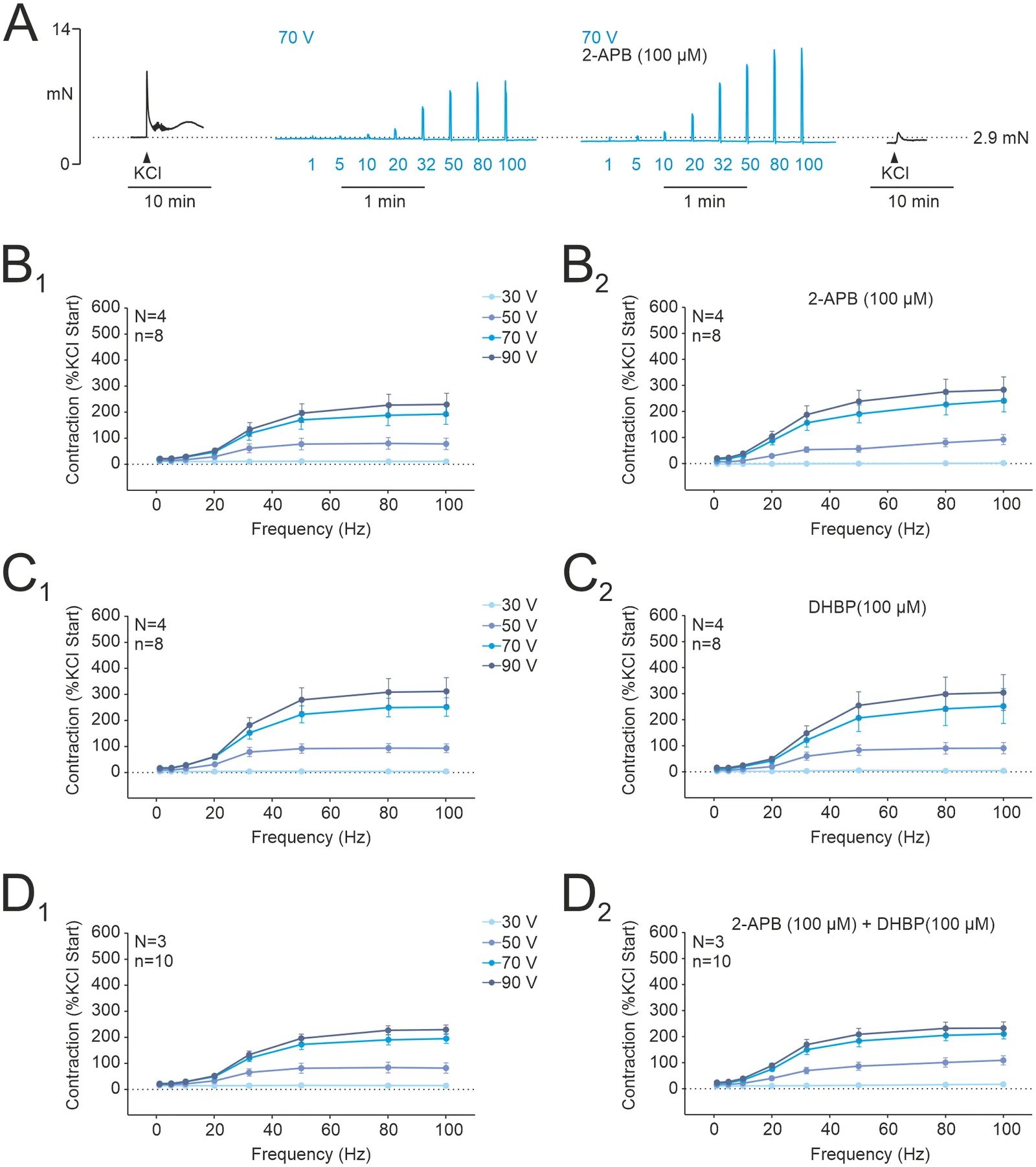
**(A)** Representative force recording consisting of 60 mM KCl, twice EFS (first baseline, then under 2-APB), and again 60 mM KCl. **(B)** Frequency-response curves for different voltages (30, 50, 70 and 90 V) either before **(B_1_)** or after 2-APB **(B_2_)**. **(C)** Frequency-response curves for different voltages (30, 50, 70 and 90 V) either before **(C_1_)** or after DHBP **(C_2_)**. **(D)** Frequency-response curves for different voltages (30, 50, 70 and 90 V) either before **(D_1_)** or after combined application of 2-APB and DHBP **(D_2_)**. Blue numbers indicate the frequency that was used. N refers to the number of animals, while n denotes the number of esophageal segments used in the experiments.

Muscarinic receptor activation also leads to Ca^2+^ release from internal stores via inositol trisphosphate (IP_3_) receptors (Harnett and Biancani, 2003; Olson et al., 2012). To test this pathway, we applied the IP_3_ receptor blocker 2-APB (100 µM, N=4, n=8, **Figures 3A, B**) and found that EFS-induced contraction even tended to be larger under these conditions. The treatment effect was statistically significant (P<0.001, 3-way ANOVA with Holm-Sidak test), and again, the effect size was moderate (-0.56). Since Ca^2+^ release from internal stores may also contribute to muscle contraction (Kirschstein et al., 2009), we used the ryanodine receptor blocker DHBP (100 µM, N=4, n=8, **Figure 3C**) to test this pathway directly. DHBP failed to reduce EFS-induced contraction. The treatment effect by this compound was not statistically significant (P=0.074, 3-way ANOVA with Holm-Sidak test) and had a small effect size (0.28). We then combined both compounds (2-APB + DHBP, N=3, n=10, **Figure 3D**) and found a statistically significant treatment effect (P<0.001, 3-way ANOVA with Holm-Sidak test, but with a negligible effect size (-0.17).

In summary, we obtained evidence that Ca^2+^ release from internal stores might have a minor role. This finding should be interpreted with caution. In our study, we used high concentrations. It is noteworthy that 2-APB has various side effects at higher concentrations, for example on gap junction channels (Bai et al., 2006), TRP channels (Hu et al., 2004), or SERCA (Bilmen et al., 2002). Inhibition of SERCA may have led to an overestimation of our results, and internal Ca²^+^ release may also play a role. Nevertheless, in our hands, despite these potential off-target effects, our findings argue against a role for IP_3_ receptors involvement.

### 3.3 Investigation of external Ca2+ influx and downstream signaling pathways

**Figure 4:**
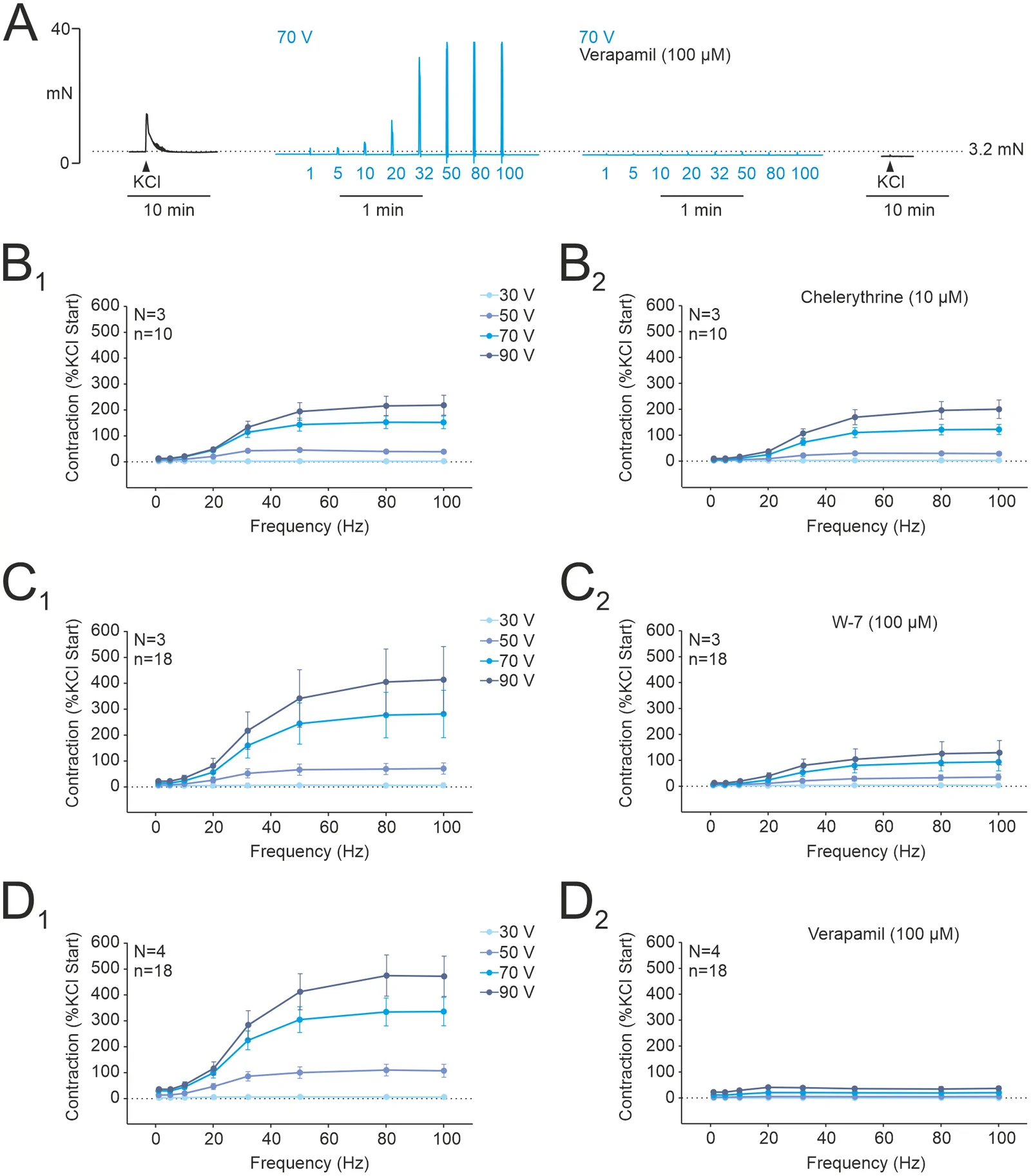
**(A)** Representative force recording consisting of 60 mM KCl, twice EFS (first baseline, then under verapamil), and again 60 mM KCl. **(B)** Frequency-response curves for different voltages (30, 50, 70 and 90 V) either before **(B_1_)** or after chelerythrine **(B_2_)**. **(C)** Frequency-response curves for different voltages (30, 50, 70 and 90 V) either before **(C_1_)** or after W-7 **(C_2_)**. **(D)** Frequency-response curves for different voltages (30, 50, 70 and 90 V) either before **(D_1_)** or after verapamil **(D_2_)**. Blue numbers indicate the frequency that was used. N refers to the number of animals, while n denotes the number of esophageal segments used in the experiments.

We next assessed the protein kinase C (PKC) pathway by applying the inhibitor chelerythrine (10 µM, N=3, n=10, **Figure 4B**). As shown in **Figure 4B**, in our animal-based analysis, chelerythrine significantly reduced the EFS-induced contraction (P<0.001, 3-way ANOVA with Holm-Sidak test), and the effect size was strongly more pronounced (5.43) than in the previous tested conditions. Thus, we concluded that PKC activation was required for EFS-induced contraction.

We then evaluated W-7 (100 µM, N=3, n=18, **Figure 4C**), a calmodulin antagonist, to interfere with Ca^2+^/calmodulin-dependent pathways. W-7 significantly reduced EFS-induced contraction (P<0.001, 3-way ANOVA with Holm-Sidak test), and the effect size (2.50) was high.

We also co-applied chelerythrine and W-7 (cheleythrine + W-7, N=3, n=10) and obtained a relative high effect size of 1.76 (versus baseline, P<0.001, 3-way ANOVA with Holm-Sidak test).

W-7 may also exhibit inhibitory effects at gap junctions (Blödow et al., 2003) or the PKC (O'Brian and Ward, 1989), albeit with a high IC_50_ value of 260 µM (O'Brian and Ward, 1989), and furthermore it might interfere with carbachol and KCl-induced contraction (Asano, 1989). We therefore performed a re-analysis to directly compare chelerythrine and W-7, respectively, with the combination of both: We observed that this combination (chelerythrine + W-7) was less effective chelerythrine alone (P<0.001, 3-way ANOVA with Holm-Sidak test; mean effect size 1.50). Therefore, under These conditions, an additional contribution of calmodulin inhibition to the suppression of contraction is likely to be minor, as supported by the comparatively large effect size observed with chelerythrine (5.43). Furthermore, the combination was less effective than W-7 alone (P<0.001, 3-way ANOVA with Holm-Sidak test; effect size -1.56). This, in fact, could imply that W-7 had occluded the chelerythrine effect.

To further identify the possible route of Ca^2+^ increase in EB muscle cells, we applied verapamil (100 µM, N=4, n=18, **Figures 4A, D**) to block L-type Ca^2+^ channels. Under our conditions, EFS-induced contractions were indeed abolished (P<0.001, 3-way ANOVA with Holm-Sidak test) resulting in a high effect size (4.26). Importantly, verapamil at this concentration may also block voltage-dependent Na^+^ channels (Norris and Bradford, 1985) and the almost abolished EFS-contraction cannot unequivocally be attributed to L-type channels only. However, this does not argue against external Ca^2+^ as the primary source in general.

It is worth mentioning that the segment-based analysis (rather than the animal-based analysis) yielded a lower effect size (0.70) for chelerythrine (**Supplementary Table 2**). This may indicate that PKC plays a different role in the proximal, middle, and distal segments. Thus, we have attributed signaling pathways with high effect sizes to the three different segments of the esophagus. While there was no statistically significant overall effect of the esophageal segments on *C_max_*, we found a tendency of chelerythrine and W-7 to affect more the distal segment and verapamil to affect more the proximal one. However, statistical significance was not achieved (P=0.05 for chelerythrine, see **Supplementary Table 1**).

In summary, we have obtained evidence that external Ca²^+^ and PKC may be necessary in EFS-induced contraction. Again, like our investigation of internal Ca^2+^ release, the results should be interpreted with caution because of the high concentration we used for verapamil and W-7. The inhibition of sodium channels by verapamil and the possible inhibition of PKC by W-7 could also contribute to an overestimation of our results.

## 4 Discussion

### 4.1 Protocol- and species-specific EFS-induced contraction

This study aimed to investigate mechanisms of EFS-elicited EB contraction. Interpretation of EFS requires distinguishing (1) stimulation site (vagus nerve vs. direct EB), (2) muscle type (striated vs. smooth), (3) contraction direction (longitudinal vs. circular), and (4) animal model. In rodents, direct EB stimulation induces a monophasic twitch-like contraction (Storr et al., 2001; Shiina et al., 2012; Boudaka et al., 2007), whereas vagal nerve stimulation induces a biphasic response when contraction was measured in the longitudinal direction (Shiina et al., 2015; Storr et al., 2001): an immediate twitch-like contraction during stimulation followed by a delayed, sustained contraction after stimulus termination. The first phase was attributed to the striated muscle in the muscularis propria, while the second phase was attributed to the smooth muscle in the mucosa. In general, atropine has no effect on direct EB stimulation (Storr et al., 2001; Shiina et al., 2012; Boudaka et al., 2007), but blocks the second phase after vagal nerve stimulation while preserving the first (Storr et al., 2001; Shiina et al., 2015). On the other hand, EFS of the vagus nerve resulted in a monophase twitch-like contraction when contraction was measured in a circular direction (Shiina et al., 2012; Boudaka et al., 2007). These studies only addressed the striated muscles of the muscularis propria (longitudinally oriented muscles of were not measured), and its contraction resembled the direct EFS-induced effect of the EB: A twitch-like contraction that was not blocked by atropine (Storr et al., 2001; Shiina et al., 2012; Boudaka et al., 2007). In our study, direct EB stimulation in longitudinal direction induced a twitch-like, atropine-insensitive, contraction. Thus, our data indicate that EFS-induced contractions might not directly arise from vagal stimulation, comparable to the data from Storr et al (Storr et al., 2001). In addition, we showed that Y-27632 had only little effect suggesting that Rho kinase might not be involved in EFS-induced contraction. Moreover, as Rho kinase is involved in carbachol-induced contraction of human LES (Sims et al., 2008), feline distal EB (Park et al., 2009c) or rat EB (unpublished observations, see **Supplementary Figure 3**), we suggest that EFS-induced contractions and cholinergic agonist-induced esophageal contractions do not share the same signaling pathways.

Besides variation in protocols hitherto used, EFS-induced contraction has been tested in different species. Our results in rats may contradict previous data on EFS in cats and pigs: Park et al (Park et al., 2009a, Park et al., 2009b, Park et al., 2010, Park et al., 2009c) demonstrated in feline distal esophageal smooth muscle that both atropine and Y-27632 block EFS-induced contractions. Lecea et al (Lecea et al., 2012) similarly reported in porcine proximal and distal esophageal smooth muscle that atropine blocks EFS-induced contractions. Worth mentioning is the study by Nakamori et al (Nakamori et al., 2012): They reported that in neonatal animals, the muscularis propria is composed of both smooth and striated muscle, whereas in adult rats, only striated muscle can be found. One may argue that we had obtained our data from rats of a wide age range, but our data on chelerythrine and W-7 were obtained from 7-8 months old animals only, so definitely adult. The feline esophagus offers the opportunity of a rather human-like composition (Park et al., 2009c) of proximal striated and distal smooth muscle (Park et al., 2010, Park et al., 2009b), while in the rat, striated muscle reached down to the distal esophagus (Gruber, 1978). Indeed, histological studies described the presence of striated muscle in the distal esophagus (Di Natale et al., 2022) and in the proximal esophagus (Su et al., 2011), and that the entire rat esophagus contains striated muscle in the propria muscularis (Nakamori et al., 2012), but there is also smooth muscle in the mucosa (Storr et al., 2000). Indeed, a transformation during aging from smooth muscle to striated muscle was reported in a review (Neuhuber and Wörl, 2016). Consistent with this anatomical issue, we did not find significant differences between proximal, middle and distal segments in our experiments (**Supplementary Table 1**) regarding c_max_ reduction. Furthermore, the EFS-induced contractions differed slightly between proximal, middle and distal segments (**Supplementary Figure 2**). Although these differences reached statistical significance (three-way ANOVA with Holm-Sidak test), the effect sizes were rather small. Differences in the histological and immunohistochemical composition of the esophagus in rats have been reported (Wörl et al., 1994), particularly in Wistar rats (Kuramoto et al., 2019). They demonstrated regional differences in the number of intrinsic neurons and the innervation of striated muscles in the rat esophagus. Thus, we did not perform histological verification of the striated muscle tissue, nor did we perform immunohistochemistry. It is a relevant limitation that the rat distal esophageal segment is less appropriate to model the human situation, but the upper two thirds of human esophagus are composed of striated muscle where data from rat esophagus can help understand the involved mechanisms, e.g. when dealing with the consequences of thoracic radiotherapy (Frerker et al., 2022; Sasso et al., 2008; Türkölmez et al., 2005; Brandt-Mainz et al., 2001).

### 4.2 Different Ca^2+^ sources for esophageal contraction.

By directly comparing circular EB and LES muscle strips, it was demonstrated in circular EB that acetylcholine activated M_2_ receptors leading to pertussis toxin-sensitive G-protein activation and diacylglycerol formation, but not to IP_3_ production and subsequent Ca^2+^ release (Sohn et al., 1993). In contrast, there is evidence that IP_3_-mediated Ca^2+^ release was an important Ca^2+^ source for LES contraction (Sohn et al., 1993; Biancani et al., 1997; Kang et al., 2001). Moreover, at least in the cat, different substructures within the LES may use either Ca^2+^ release or Ca^2+^ influx for acetylcholine-induced contractions (Muinuddin et al., 2004). On the other hand, L-type Ca^2+^ channel blocker inhibited significantly the EFS-induced contraction in distal feline esophagus (Park et al., 2009a).

Our findings suggest that the increase in intracellular Ca²^+^ required for muscle contraction may arise from extracellular Ca²^+^ influx rather than from the release of Ca²^+^ from intracellular stores. However, because the inhibitors were used at very high concentrations, these conclusions should be interpreted with caution.

First, the available literature on verapamil in the context of organ bath experiments describes a wide range of concentrations. In other organ bath studies, a verapamil concentration of 1µM (Wani et al., 2018), 10 µM (Liao et al., 2000; Mustafa and Thulesius, 2001), 40µM (Kirschstein et al., 2014, Kirschstein et al., 2009), or even 100µM (Mader et al., 2016; Ortiz et al., 1988; Huang et al., 2011; Burchés et al., 1992) was applied. In our study, we used 100 µM verapamil. As already mentioned in the results section, verapamil may also block voltage-dependent Na+ channels (Norris and Bradford, 1985) at this concentration. Thus, the almost abolished EFS-contraction cannot unequivocally be attributed to L-type channels only. For comparison, experiments using a sodium channel blocker, such as tetrodotoxin (TTX), would be helpful. We did not use sodium channel blockers in our study. Therefore, it may not be possible to distinguish with certainty between vagal stimulation and direct muscle stimulation. Since our findings regarding the effect of atropine on EFS are consistent with those described by other authors, we consider vagal stimulation unlikely and assume that direct muscle stimulation is the underlying mechanism. We note that Storr et al (Storr et al., 2001) incubated the rat esophagus with atropine and TTX, and directly stimulated the esophagus using EFS. Atropine had no effect, whereas tetrodotoxin blocked contraction. Since we used a similar experimental setup, we did not use sodium channel blockers in our experiments.

Second, as already mentioned in the results section, higher concentrations of 2-APB can inhibit SERCA (Bilmen et al., 2002). SERCA pumps calcium from the cytosol of the muscle cell back into the endoplasmic reticulum (Arruda et al., 2007). In a recently initiated study, we were able to show that the SERCA inhibitor cyclopiazonic acid (CPA (Yao et al., 2011)) causes a leftward shift in EFS-induced contractile force in the esophageal muscle of rats (**Supplementary Figure 4** A+B, unpublished own data). This may suggest that intracellular calcium plays a role in EFS-induced contraction. However, in the present study, there was no leftward shift. The use of other inhibitors, such as xestospongin C for IP_3_ receptors and ryanodine for RyR, would be useful for future analyses.

As shown in **Figure 4A** (and **Supplementary Figure 1B**), the KCl End response was reduced compared to KCl Start. This is particularly true of esophageal segments treated with verapamil. On the one hand, this could be due to poor verapamil wash-out. On the other hand, we could observe that repeated application of carbachol only led to small tachyphylaxis, but the KCl End response was also reduced, although verapamil had not been tested (**Supplementary Figure 5**, unpublished own data). Therefore, in our view, a deterioration of the organ over time appears to be more relevant than poor clearance of verapamil. The deterioration of the organ could also be one reason why the treatment effect (3-way ANOVA) yielded predominantly statistically significant differences.

Finaly, when interpreting the data we obtained, it is important to note that the analyses performed are animal-based. In **Supplementary Table 2**, we compare segment-based statistics with animal-based statistics. It is noteworthy that the effect size of chelerythrine and verapamil is greater based on the animal-based statistics presented in the manuscript, and that the small number of animals (N) contributed to weaker statistical results, which could explain this difference.

Based on our findings with 2-APB, DHBP, and verapamil, extracellular Ca²^+^ appears to be the primary source driving EFS-induced EB contractions. However, given the potential off-target effects of these compounds, alternative mechanisms may have contributed to the observed responses and thus led to an overestimation of this effect. Nevertheless, these limitations do not contradict the involvement of extracellular Ca²^+^ in the contractile response. At the same time, a contribution of Ca²^+^ release from intracellular stores cannot be fully excluded.

### 4.3 Possible Ca^2+^-dependent signaling pathways of esophageal contraction.

Which signal pathways follow the intracellular Ca^2+^ increase? In the feline distal smooth muscle, chelerythrine had no significant effect on off-contraction, whereas ML-9 (MLCK inhibitor) did, suggesting that EFS-induced contraction is mediated by MLCK rather than PKC (Park et al., 2009b). On the other hand, chelerythrine as well as ML-9 inhibited on-contraction in the presence of a NO synthase inhibitor, suggesting that both, MLCK and PKC, are involved in EFS-induced contraction (Park et al., 2010). In our study, chelerythrine also inhibited the EFS-induced EB contractions. Thus, we conclude that PKC activation might be involved in EFS-induced striated muscle contraction. With respect to PKC isoforms, at present no data are available for the rat esophageal muscle. In the cat, there is some notion that PKCϵ contributed to esophageal circular muscle contraction (Shin et al., 2002). Just this isoform, however, belongs to the novel PKC isoforms that are known for their Ca^2+^-independent activation (Moussa et al., 2025). Albeit the limited notion of PKC isoforms in different muscular tissues across various species is still an unmet need, our data at least supports the view that Ca^2+^-independent novel PKC isoforms are rather unlikely to be involved in EFS-induced contraction. Moreover, our data on similar chelerythrine effects in proximal, middle and distal segments (p-value nearly significant, P=0.05, **Supplementary Table 1**) demonstrate that PKC activation in general might be involved in signaling pathway of esophageal EFS-induced contraction.

In our study, calmodulin-dependent pathways were also recruited by EFS-induced contractions as evidenced by the calmodulin antagonist W-7, which had no effect on feline acetylcholine-induced esophageal contraction (Hillemeier et al., 1991). Intriguingly, the combination of both probes, chelerythrine and W-7, was in part occlusive implying interference between Ca^2+^/calmodulin- and PKC-dependent pathways. Theoretically, Ca^2+^/calmodulin could give rise to myosin light chain kinase activation, but this was shown to be specific for feline LES contraction (Sohn et al., 2001). We believe that these findings can be most parsimoniously explained by impaired phospholipase C activation by Ca^2+^/calmodulin in the presence of W-7 (Brubaker-Purkey and Woodruff, 2013). This, in turn, would be followed by decreased diacylglycerol production and thereby reduced PKC activity, regardless whether conventional or novel isoforms (Ohno et al., 1991). Such a mechanism could explain the occlusion effect of W-7 compared to the effect of combined chelerythrine and W-7. This is reinforced by the fact that W-7 also might inhibit the PKC (O'Brian and Ward, 1989).

Based on our results with chelerythrine and W-7, the increase in intracellular calcium might be followed by activation of PKC and calmodulin. However, like our other results, this finding should be interpreted with caution. Because of possible PKC-inhibition of W-7, it is difficult to distinguish the independent contributions of calmodulin and PKC. If W-7 directly inhibits PKC at the concentration used, the observed occlusion may simply reflect pharmacological overlap rather than a shared physiological signaling mechanism.

## 5 Limitations of the study

Important limitations of our study ought to be noted: (1) We used relatively high concentrations (100 µM). Therefore, potential off-target effects and alternative mechanisms may have contributed to the observed responses, possibly leading to an overestimation of our results. Moreover, a contribution of Ca²^+^ release from intracellular stores cannot be fully excluded. Thus, our results should be interpreted with caution. (2) We did not use sodium channel blockers in our study (for example TTX). Consequently, it may not be possible to distinguish with certainty between vagal stimulation and direct muscle stimulation, and our results obtained with verapamil might be an overestimation. (3) In addition: Assuming that EFS can activate terminal nerve endings, testing alternative signaling pathways (purinergic, tachykininergic, or other non-cholinergic pathways) is a promising approach. In our current study, the focus was laid on identifying the source of calcium; therefore, we recommend testing alternative signaling pathways in subsequent studies on the striated muscle of the rat esophagus. (4) One could argue that the inhibitors for internal Ca^2+^ release we used generally have no effect, and that is why no effect was observed. The use of other inhibitors (e.g., IP_3_ inhibitor xestospongin C or RyR inhibitor ryanodine) might have provided clarity. In the **Supplementary Figure 4**, for example, we show a left shift in contractile force following incubation with CPA, a SERCA inhibitor. This suggests that intracellular calcium does indeed play a role. (5) In our study, the verapamil-treated preparations exhibited the lowest KCl End response, raising the possibility of reduced tissue responsiveness following prolonged exposure. This could indicate that the high concentration of verapamil we used had a negative effect on the tissue. However, in organ bath experiments, tissue aging frequently occurs, leading to reduced response rates. In **Supplementary Figure 5**, we show an example in which KCl was administered before and after repetitive stimulation with carbachol. It can be seen that the maximum contraction force decreases over the course of the experiment.

## 6 Conclusions

Based on our findings with 2-APB, DHBP, and verapamil, extracellular Ca²^+^ appears to be the source of the increase in intracellular Ca²^+^ underlying EFS-induced EB contractions. Furthermore, the effects observed with chelerythrine and W-7 may suggest that this increase in intracellular Ca²^+^ may subsequently activate PKC- and calmodulin-dependent signaling pathways.

## Data Availability

The raw data supporting the conclusions of this article will be made available by the authors, without undue reservation.

## Supplementary material

Supplementary Figure 1 **(A)** Baseline tone in all 11 experimental groups. The total mean (2.8 mN) is shown as a gray box in the background. Asterisks denote significant differences to this mean value (t-test). **(B)** Percentage of the high KCl response at the end of the experiment (KCl End) to the first KCl response (KCl Start) in all 11 experimental groups. The total mean (64%) is shown as a gray box. Asterisks denote significant differences to 100% (dotted line). N refers to the number of animals, while n denotes the number of esophageal segments used in the experiments.

Supplementary Figure 2 Frequency-dependent contraction induced by electrical field stimulation (EFS) is shown for stimulus intensities of 30 V, 50 V, 70 V and 90 V, separately for the proximal portion **(A)**, the middle portion **(B)** and the distal portion **(C)**. Three-way ANOVA (factors stimulus intensity, frequency, location) with Holm-Sidak posthoc test detected a significant effect of location: proximal versus middle (P<0.01), proximal versus distal (P<0.05), middle versus distal (P<0.05). N refers to the number of animals, while n denotes the number of esophageal segments used in the experiments. However, the effect sizes were rather small (proximal versus middle: 0.37, proximal versus distal: 0.20, middle versus distal: 0.17).

Supplementary Figure 3 The addition of carbachol (c1) induces a long-lasting contraction of the esophagus, which is terminated by a flushing procedure to fresh the organ bath solution (w). The second contraction (c2) after the addition of Y-27632 (+) has a lower amplitude. Pre-incubation time with the inhibitor Y-27632 was 10 minutes.

Supplementary Figure 4 Comparison of EFS-induced contractions of different amplitudes and frequencies before **(A)** and after **(B)** incubation with the SERCA inhibitor CPA. A leftward shift in contractile force is observed. x-axis: Frequency (Hz), y-axis: Contractile force (mN).

Supplementary Figure 5 Repetitive immediately carbachol-induced (c) contractions that were terminated by refreshing the organ bath solution (w). It can be seen that the maximum contraction strength decreases over time. The second KCl response (k2) also showed a weaker contraction compared to the first KCl response (k1).

Supplementary Table 1 Comparison between proximal, middle and distal segments of the esophagus (segment-based analysis). Data are given as mean ± SEM. Statistical comparison was performed using Kruskal-Wallis test. SEM, standard error of the mean; n, number of segments; c _max_, maximum contraction.

Supplementary Table 2 Comparison between segment-based and animal-based analysis for treatment effect and effect size. Effect size was calculated based on absolute EFS-mediated contractions.

## References

[B1] Ackbar R. Malvasio V. Holzer P. Saxena A. K. (2012). *In vitro* effect of bethanechol and suberyldicholine on regions of Guinea pig esophagus. J. Surg. Res. 174, 56–61. doi: 10.1016/j.jss.2010.11.899 21227463

[B2] Akbarali H. I. Bieger D. Ohia S. E. Triggle C. R. (1988a). Similarity of relaxations evoked by BRL 34915, pinacidil and field-stimulation in rat oesophageal tunica muscularis mucosae. Br. J. Pharmacol. 95, 519–525. doi: 10.1111/j.1476-5381.1988.tb11672.x 3228674 PMC1854173

[B3] Akbarali H. I. Bieger D. Triggle C. R. (1988b). Inhibition of field stimulation-evoked relaxations in rat oesophageal smooth muscle by the calcium antagonist PN 200-110. Br. J. Pharmacol. 95, 512–518. doi: 10.1111/j.1476-5381.1988.tb11671.x 2976289 PMC1854195

[B4] Anderson C. D. Kendig D. M. Al-Qudah M. Mahavadi S. Murthy K. S. Grider J. R. (2014). Role of various kinases in muscarinic M3 receptor-mediated contraction of longitudinal muscle of rat colon. J. Smooth. Muscle Res. 50, 103–119. doi: 10.1540/jsmr.50.103 25891767 PMC4862207

[B5] Arruda A. P. Nigro M. Oliveira G. M. Meis L. (2007). Thermogenic activity of Ca2+-ATPase from skeletal muscle heavy sarcoplasmic reticulum: the role of ryanodine Ca2+ channel. Biochim. Biophys. Acta 1768, 1498–1505. doi: 10.1016/j.bbamem.2007.03.016 17466935

[B6] Asano M. (1989). Divergent pharmacological effects of three calmodulin antagonists, N-(6-aminohexyl)-5-chloro-1-naphthalenesulfonamide (W-7), chlorpromazine and calmidazolium, on isometric tension development and myosin light chain phosphorylation in intact bovine tracheal smooth muscle. J. Pharmacol. Exp. Ther. 251, 764–773. doi: 10.1016/s0022-3565(25)20876-x 2810127

[B7] Bai D. Del Corsso C. Srinivas M. Spray D. C. (2006). Block of specific gap junction channel subtypes by 2-aminoethoxydiphenyl borate (2-APB). J. Pharmacol. Exp. Ther. 319, 1452–1458. doi: 10.1124/jpet.106.112045 16985167

[B8] Bai X. Ihara E. Hirano K. Tanaka Y. Nakano K. Kita S. . (2018). Endogenous hydrogen sulfide contributes to tone generation in porcine lower esophageal sphincter via Na+/Ca2+ exchanger. Cell. Mol. Gastroenterol. Hepatol. 5, 209–221. doi: 10.1016/j.jcmgh.2017.11.004 29379856 PMC5782486

[B9] Bai X. Ihara E. Otsuka Y. Tsuruta S. Hirano K. Tanaka Y. . (2019). Involvement of different receptor subtypes in prostaglandin E2-induced contraction and relaxation in the lower esophageal sphincter and esophageal body. Eur. J. Pharmacol. 857, 172405. doi: 10.1016/j.ejphar.2019.172405 31128092

[B10] Biancani P. Sohn U. D. Rich H. G. Harnett K. M. Behar J. (1997). Signal transduction pathways in esophageal and lower esophageal sphincter circular muscle. Am. J. Med. 103, 23S–28S. doi: 10.1016/s0002-9343(97)00316-1 9422618

[B11] Bilmen J. G. Wootton L. L. Godfrey R. E. Smart O. S. Michelangeli F. (2002). Inhibition of SERCA Ca2+ pumps by 2-aminoethoxydiphenyl borate (2-APB). 2-APB reduces both Ca2+ binding and phosphoryl transfer from ATP, by interfering with the pathway leading to the Ca2+-binding sites. Eur. J. Biochem. 269, 3678–3687. doi: 10.1046/j.1432-1033.2002.03060.x 12153564

[B12] Blödow A. Ngezahayo A. Ernst A. Kolb H.-A. (2003). Calmodulin antagonists suppress gap junction coupling in isolated Hensen cells of the Guinea pig cochlea. Pflugers. Arch. 446, 36–41. doi: 10.1007/s00424-002-1004-9 12690460

[B13] Boudaka A. Wörl J. Shiina T. Neuhuber W. L. Kobayashi H. Shimizu Y. . (2007). Involvement of TRPV1-dependent and -independent components in the regulation of vagally induced contractions in the mouse esophagus. Eur. J. Pharmacol. 556, 157–165. doi: 10.1016/j.ejphar.2006.11.005 17156774

[B14] Brandt-Mainz K. Mallek D. Pöttgen C. Eising E. G. Bockisch A. Stuschke M. . (2001). Parametric oesophageal multiple swallow scintigraphy for validation of dysphageal symptoms during external beam irradiation of mediastinal tumours. Eur. J. Nucl. Med. 28, 313–319. doi: 10.1007/s002590000453 11315598

[B15] Brubaker-Purkey B. J. Woodruff R. I. (2013). Vitellogenesis in the fruit fly, Drosophila melanogaster: antagonists demonstrate that the PLC, IP3/DAG, PK-C pathway is triggered by calmodulin. J. Insect Sci. 13, 68. doi: 10.1673/031.013.6801 24228869 PMC3835028

[B16] Burchés E. Cortijo J. Orón J. D. Ortiz J. L. Morcillo E. J. (1992). Effects of calcium antagonists on rat normal and skinned fundus. J. Pharm. Pharmacol. 44, 500–506. doi: 10.1111/j.2042-7158.1992.tb03654.x 1359075

[B17] Cao W. Chen Q. Sohn U. D. Kim N. Kirber M. T. Harnett K. M. . (2001). Ca2+-induced contraction of cat esophageal circular smooth muscle cells. Am. J. Physiol. Cell Physiol. 280, C980–C992. doi: 10.1152/ajpcell.2001.280.4.C980 11245615

[B18] Capanoglu D. Coskunsever D. Olukman M. Ülker S. Bor S. (2016). Esophageal epithelial resistance and lower esophageal sphincter muscle contraction increase in a chronic diabetic rabbit model. Dig. Dis. Sci. 61, 1879–1887. doi: 10.1007/s10620-016-4111-8 26972084

[B19] Darios E. S. Winner B. M. Charvat T. Krasinksi A. Punna S. Watts S. W. (2016). The adipokine chemerin amplifies electrical field-stimulated contraction in the isolated rat superior mesenteric artery. Am. J. Physiol. Heart Circ. Physiol. 311, H498–H507. doi: 10.1152/ajpheart.00998.2015 27371688 PMC5008655

[B20] Di Natale M. R. Patten L. Molero J. C. Stebbing M. J. Hunne B. Wang X. . (2022). Organisation of the musculature of the rat stomach. J. Anat. 240, 711–723. doi: 10.1111/joa.13587 34747011 PMC8930815

[B21] Frerker B. Fiedler S. Kirschstein T. Lange F. Porath K. Sellmann T. . (2022). Effects of microbeam irradiation on rodent esophageal smooth muscle contraction. Cells 12(1), 176. doi: 10.3390/cells12010176 36611969 PMC9818134

[B22] Ghasemi-Kasman M. Dehpour A. R. Mani A. R. (2012). D-serine modulates non-adrenergic non-cholinergic contraction of lower esophageal sphincter in rats. Eur. J. Pharmacol. 696, 155–160. doi: 10.1016/j.ejphar.2012.09.011 23022330

[B23] González A. A. Farré R. Clavé P. (2004). Different responsiveness of excitatory and inhibitory enteric motor neurons in the human esophagus to electrical field stimulation and to nicotine. Am. J. Physiol. Gastrointest. Liver. Physiol. 287, G299–G306. doi: 10.1152/ajpgi.00534.2003 15016616

[B24] González A. A. Farré R. Monés J. Capellà G. Clavé P. (2000). Pharmacological and molecular characterization of muscular cholecystokinin receptors in the human lower oesophageal sphincter. Neurogastroenterol. Motil. 12, 539–546. doi: 10.1046/j.1365-2982.2000.00229.x 11123709

[B25] Goyal R. K. Chaudhury A. (2008). Physiology of normal esophageal motility. J. Clin. Gastroenterol. 42, 610–619. doi: 10.1097/MCG.0b013e31816b444d 18364578 PMC2728598

[B26] Gruber H. (1978). Motor innervation of the striated oesophagus muscle. Part 1. Intramural distribution of the right and left vagus nerve in the rat oesophagus as revealed by the glycogen depletion technique. J. Neurol. Sci. 36, 41–53. doi: 10.1016/0022-510x(78)90160-0 650254

[B27] Hançerlioğullari O. Cakmak M. Soyer T. Aktuna Z. (2012). *In vitro* sensitivity of rat esophagus to agonists in different alkaline mediums. J. Pediatr. Surg. 47, 1848–1852. doi: 10.1016/j.jpedsurg.2012.04.020 23084196

[B28] Harnett K. M. Biancani P. (2003). Calcium-dependent and calcium-independent contractions in smooth muscles. Am. J. Med. 115, 24S–30S. doi: 10.1016/s0002-9343(03)00232-8 12928071

[B29] Harnett K. M. Cao W. Biancani P. (2005). Signal-transduction pathways that regulate smooth muscle function I. Signal transduction in phasic (esophageal) and tonic (gastroesophageal sphincter) smooth muscles. Am. J. Physiol. Gastrointest. Liver. Physiol. 288, G407–G416. doi: 10.1152/ajpgi.00398.2004 15701619

[B30] Hillemeier C. Bitar K. N. Marshall J. M. Biancani P. (1991). Intracellular pathways for contraction in gastroesophageal smooth muscle cells. Am. J. Physiol. 260, G770–G775. doi: 10.1152/ajpgi.1991.260.5.G770 2035645

[B31] Hu H.-Z. Gu Q. Wang C. Colton C. K. Tang J. Kinoshita-Kawada M. . (2004). 2-aminoethoxydiphenyl borate is a common activator of TRPV1, TRPV2, and TRPV3. J. Biol. Chem. 279, 35741–35748. doi: 10.1074/jbc.M404164200 15194687

[B32] Huang K.-H. Chang C.-C. Ho J.-D. Lu R.-H. Tsai L. H. (2011). Role of taurine on acid secretion in the rat stomach. J. Biomed. Sci. 18, 11. doi: 10.1186/1423-0127-18-11 21294907 PMC3042912

[B33] Hurley B. R. Preiksaitis H. G. Sims S. M. (1999). Characterization and regulation of Ca2+-dependent K+ channels in human esophageal smooth muscle. Am. J. Physiol. 276, G843–G852. doi: 10.1152/ajpgi.1999.276.4.G843 10198326

[B34] Igarashi-Hisayoshi Y. Ihara E. Bai X. Higashi C. Ikeda H. Tanaka Y. . (2023). Determination of region-specific roles of the M3 muscarinic acetylcholine receptor in gastrointestinal motility. Dig. Dis. Sci. 68, 439–450. doi: 10.1007/s10620-022-07637-y 35947306

[B35] Kang H. Y. Lee T. S. Lee Y. P. Lee D. W. La H. O. Song H. J. . (2001). Interaction of calmodulin- and PKC-dependent contractile pathways in cat lower esophageal sphincter (LES). Arch. Pharm. Res. 24, 546–551. doi: 10.1007/BF02975163 11794533

[B36] Kim N. Cao W. Song I. S. Kim C. Y. Harnett K. M. Cheng L. . (2004). Distinct kinases are involved in contraction of cat esophageal and lower esophageal sphincter smooth muscles. Am. J. Physiol. Cell Physiol. 287, C384–C394. doi: 10.1152/ajpcell.00390.2003 15128504

[B37] Kirschstein T. Protzel C. Porath K. Sellmann T. Köhling R. Hakenberg O. W. (2014). Age-dependent contribution of Rho kinase in carbachol-induced contraction of human detrusor smooth muscle *in vitro*. Acta Pharmacol. Sin. 35, 74–81. doi: 10.1038/aps.2013.126 24122009 PMC4075738

[B38] Kirschstein T. Rehberg M. Bajorat R. Tokay T. Porath K. Köhling R. (2009). High K+-induced contraction requires depolarization-induced Ca2+ release from internal stores in rat gut smooth muscle. Acta Pharmacol. Sin. 30, 1123–1131. doi: 10.1038/aps.2009.98 19578389 PMC4006678

[B39] Koc A. Koc D. S. Askin C. I. Kara H. Ozturk Fincan G. S. Ozger Ilhan S. . (2024). Effects of hydrogen sulfide on relaxation responses in the lower esophageal sphincter in rabbits: the potential role of potassium channels. Naunyn Schmiedebergs Arch. Pharmacol. 397, 1537–1550. doi: 10.1007/s00210-023-02695-z 37668686

[B40] Kuramoto H. Kadowaki M. Yoshida N. (2013). Morphological demonstration of a vagal inhibitory pathway to the lower esophageal sphincter via nitrergic neurons in the rat esophagus. Neurogastroenterol. Motil. 25, e485-94. doi: 10.1111/nmo.12146 23634870

[B41] Kuramoto H. Yoshimura R. Sakamoto H. Kadowaki M. (2019). Regional variations in the number distribution of intrinsic myenteric neurons and coinnervated motor endplates on the striated muscles in the rat esophagus. Auton. Neurosci. 219, 25–32. doi: 10.1016/j.autneu.2019.03.004 31122598

[B42] Lecea B. Gallego D. Farré R. Clavé P. (2012). Origin and modulation of circular smooth muscle layer contractions in the porcine esophagus. Neurogastroenterol. Motil. 24, 779–789. doi: 10.1111/j.1365-2982.2012.01936.x 22632463

[B43] Lecea B. Gallego D. Farré R. Opazo A. Aulí M. Jiménez M. . (2011). Regional functional specialization and inhibitory nitrergic and nonnitrergic coneurotransmission in the human esophagus. Am. J. Physiol. Gastrointest. Liver. Physiol. 300, G782–G794. doi: 10.1152/ajpgi.00514.2009 21330444

[B44] Liao J. W. Kang J. J. Liu S. H. Jeng C. R. Cheng Y. W. Hu C. M. . (2000). Effects of cartap on isolated mouse phrenic nerve diaphragm and its related mechanism. Toxicol. Sci. 55, 453–459. doi: 10.1093/toxsci/55.2.453 10828278

[B45] Mader F. Krause L. Tokay T. Hakenberg O. W. Köhling R. Kirschstein T. (2016). P2Y receptor-mediated transient relaxation of rat longitudinal ileum preparations involves phospholipase C activation, intracellular Ca(2+) release and SK channel activation. Acta Pharmacol. Sin. 37, 617–628. doi: 10.1038/aps.2015.137 27018177 PMC4857539

[B46] Morishita R. Yoshimura R. Sakamoto H. Kuramoto H. (2023). Localization of substance P (SP)-immunoreactivity in the myenteric plexus of the rat esophagus. Histochem. Cell Biol. 159, 7–21. doi: 10.1007/s00418-022-02104-1 35507035

[B47] Moussa H. Y. A. Shin K. C. Park Y. (2025). Ca2+/calmodulin and protein kinase C (PKC) reverse the vesicle fusion arrest by unmasking PIP2. Sci. Adv. 11, eadr9859. doi: 10.1126/sciadv.adr9859 40009675 PMC11864169

[B48] Muinuddin A. Neshatian L. Gaisano H. Y. Diamant N. E. (2004). Calcium source diversity in feline lower esophageal sphincter circular and sling muscle. Am. J. Physiol. Gastrointest. Liver. Physiol. 286, G271–G277. doi: 10.1152/ajpgi.00291.2003 14563670

[B49] Muinuddin A. Paterson W. G. (2001). Initiation of distension-induced descending peristaltic reflex in opossum esophagus: role of muscle contractility. Am. J. Physiol. Gastrointest. Liver. Physiol. 280, G431–G438. doi: 10.1152/ajpgi.2001.280.3.G431 11171625

[B50] Murthy K. S. (2006). Signaling for contraction and relaxation in smooth muscle of the gut. Annu. Rev. Physiol. 68, 345–374. doi: 10.1146/annurev.physiol.68.040504.094707 16460276

[B51] Mustafa S. M. Pilcher C. W. Williams K. I. (1999). Cooling-induced bronchoconstriction: the role of ion-pumps and ion-carrier systems. Pharmacol. Res. 39, 125–136. doi: 10.1006/phrs.1998.0412 10072703

[B52] Mustafa S. M. Thulesius O. (2001). Cooling-induced gastrointestinal smooth muscle contractions in the rat. Fundam. Clin. Pharmacol. 15, 349–354. doi: 10.1046/j.1472-8206.2001.00034.x 11903504

[B53] Nakamori C. Shiina T. Shimizu Y. (2012). Postnatal changes in vagal control of esophageal muscle contractions in rats. Life Sci. 90, 495–501. doi: 10.1016/j.lfs.2012.01.004 22285836

[B54] Neuhuber W. L. Wörl J. (2016). Enteric co-innervation of striated muscle in the esophagus: still enigmatic? Histochem. Cell Biol. 146, 721–735. doi: 10.1007/s00418-016-1500-1 27678007

[B55] Norris D. K. Bradford H. F. (1985). On the specificity of verapamil as a calcium channel-blocker. Biochem. Pharmacol. 34, 1953–1956. doi: 10.1016/0006-2952(85)90314-4 4004910

[B56] O'Brian C. A. Ward N. E. (1989). Binding of protein kinase C to N-(6-aminohexyl)-5-chloro-1-naphthalenesulfonamide through its ATP binding site. Biochem. Pharmacol. 38, 1737–1742. doi: 10.1016/0006-2952(89)90406-1 2735931

[B57] Oh K. H. Nam Y. Jeong J. H. Kim I. K. Sohn U. D. (2014). The effect of DA-9701 on 5-hydroxytryptamine-induced contraction of feline esophageal smooth muscle cells. Molecules 19, 5135–5149. doi: 10.3390/molecules19045135 24759073 PMC6271506

[B58] Ohno S. Akita Y. Hata A. Osada S. Kubo K. Konno Y. . (1991). Structural and functional diversities of a family of signal transducing protein kinases, protein kinase C family; two distinct classes of PKC, conventional cPKC and novel nPKC. Adv. Enzyme Regul. 31, 287–303. doi: 10.1016/0065-2571(91)90018-h 1877391

[B59] Olson M. L. Sandison M. E. Chalmers S. McCarron J. G. (2012). Microdomains of muscarinic acetylcholine and Ins(1,4,5)P_3_ receptors create 'Ins(1,4,5)P_3_ junctions' and sites of Ca²+ wave initiation in smooth muscle. J. Cell Sci. 125, 5315–5328. doi: 10.1242/jcs.105163 22946060 PMC3561854

[B60] Ortiz J. L. Cortijo J. Morcillo E. J. Sanz C. Perpiñá M. Esplugues J. (1988). The spasmogenic effect of caffeine in trachealis isolated from control and actively sensitized Guinea-pigs. Eur. J. Pharmacol. 158, 243–249. doi: 10.1016/0014-2999(88)90073-8 3253100

[B61] Park J. H. Kim H. S. Park S. Y. Im C. Jeong J. H. Kim I. K. . (2009a). The influences of g proteins, ca, and k channels on electrical field stimulation in cat esophageal smooth muscle. Kor. J. Physiol. Pharmacol. 13, 393–400. doi: 10.4196/kjpp.2009.13.5.393 19915703 PMC2776901

[B62] Park S. Y. Park S. U. Sohn U. D. (2009b). Regulators involved in the electrically stimulated response of feline esophageal smooth muscle. Pharmacology 84, 346–355. doi: 10.1159/000253876 19887885

[B63] Park S. Y. Shim J. H. Kim M. Sun Y. H. Kwak H. S. Yan X. . (2010). MLCK and PKC involvements via Gi and Rho A protein in contraction by the electrical field stimulation in feline esophageal smooth muscle. Kor. J. Physiol. Pharmacol. 14, 29–35. doi: 10.4196/kjpp.2010.14.1.29 20221277 PMC2835980

[B64] Park S. Y. Song H. J. Sohn U. D. (2009c). Participation of Rho-associated kinase in electrical stimulated and acetylcholine-induced contraction of feline esophageal smooth muscle. Eur. J. Pharmacol. 607, 220–225. doi: 10.1016/j.ejphar.2009.02.027 19239907

[B65] Rattan S. Puri R. N. Fan Y.-P. (2003). Involvement of rho and rho-associated kinase in sphincteric smooth muscle contraction by angiotensin II. Exp. Biol. Med. (Maywood). 228, 972–981. doi: 10.1177/153537020322800814 12968070

[B66] Saegusa Y. Takeda H. Muto S. Oridate N. Nakagawa K. Sadakane C. . (2011). Decreased motility of the lower esophageal sphincter in a rat model of gastroesophageal reflux disease may be mediated by reductions of serotonin and acetylcholine signaling. Biol. Pharm. Bull. 34, 704–711. doi: 10.1248/bpb.34.704 21532161

[B67] Sasso G. Rambaldi P. Sasso F. S. Cuccurullo V. Murino P. Puntieri P. . (2008). Scintigraphic evaluation of oesophageal transit during radiotherapy to the mediastinum. BMC Gastroenterol. 8, 51. doi: 10.1186/1471-230X-8-51 18986522 PMC2611991

[B68] Shiina T. Naitou K. Nakamori H. Sakai H. Shimizu Y. (2015). Regulation of longitudinal esophageal motility in the house musk shrew (Suncus murinus). Auton. Neurosci. 189, 37–42. doi: 10.1016/j.autneu.2015.02.003 25694232

[B69] Shiina T. Naitou K. Nakamori H. Suzuki Y. Horii K. Sano Y. . (2016). Serotonin-induced contractile responses of esophageal smooth muscle in the house musk shrew (Suncus murinus). Neurogastroenterol. Motil. 28, 1641–1648. doi: 10.1111/nmo.12863 27194102

[B70] Shiina T. Shima T. Suzuki Y. Wörl J. Shimizu Y. (2012). Neural regulation of esophageal striated muscle in the house musk shrew (Suncus murinus). Auton. Neurosci. 168, 25–31. doi: 10.1016/j.autneu.2012.01.003 22285704

[B71] Shiina T. Suzuki Y. Horii K. Sawamura T. Yuki N. Horii Y. . (2024). Purinergic inhibitory regulation of esophageal smooth muscle is mediated by P2Y receptors and ATP-dependent potassium channels in rats. J. Physiol. Sci. 74, 26. doi: 10.1186/s12576-024-00916-5 38654149 PMC11036717

[B72] Shimbo T. Adachi T. Fujisawa S. Hongoh M. Ohba T. Ono K. (2016). *In vitro* effect of nicorandil on the carbachol-induced contraction of the lower esophageal sphincter of the rat. J. Pharmacol. Sci. 131, 267–274. doi: 10.1016/j.jphs.2016.07.005 27562702

[B73] Shin C. Y. Lee Y. P. Lee T. S. Je H. D. Kim D. S. Sohn U. D. (2002). The signal transduction of endothelin-1-induced circular smooth muscle cell contraction in cat esophagus. J. Pharmacol. Exp. Ther. 302, 924–934. doi: 10.1124/jpet.302.3.924 12183648

[B74] Sims S. M. Chrones T. Preiksaitis H. G. (2008). Calcium sensitization in human esophageal muscle: role for RhoA kinase in maintenance of lower esophageal sphincter tone. J. Pharmacol. Exp. Ther. 327, 178–186. doi: 10.1124/jpet.108.140806 18628484

[B75] Sims S. M. Jiao Y. Preiksaitis H. G. (1997). Regulation of intracellular calcium in human esophageal smooth muscles. Am. J. Physiol. 273, C1679–C1689. doi: 10.1152/ajpcell.1997.273.5.C1679 9374655

[B76] Smid S. D. Blackshaw L. A. (2000). Vagal ganglionic and nonadrenergic noncholinergic neurotransmission to the ferret lower oesophageal sphincter. Auton. Neurosci. 86, 30–36. doi: 10.1016/S1566-0702(00)00210-1 11269922

[B77] Sohn U. D. Cao W. Tang D. C. Stull J. T. Haeberle J. R. Wang C. L. . (2001). Myosin light chain kinase- and PKC-dependent contraction of LES and esophageal smooth muscle. Am. J. Physiol. Gastrointest. Liver. Physiol. 281, G467–G478. doi: 10.1152/ajpgi.2001.281.2.G467 11447027

[B78] Sohn U. D. Chiu T. T. Bitar K. N. Hillemeier C. Behar J. Biancani P. (1994). Calcium requirements for acetylcholine-induced contraction of cat esophageal circular muscle cells. Am. J. Physiol. 266, G330–G338. doi: 10.1152/ajpgi.1994.266.2.G330 8141307

[B79] Sohn U. D. Harnett K. M. Petris G. Behar J. Biancani P. (1993). Distinct muscarinic receptors, G proteins and phospholipases in esophageal and lower esophageal sphincter circular muscle. J. Pharmacol. Exp. Ther. 267, 1205–1214. doi: 10.1016/s0022-3565(25)39543-1 8263781

[B80] Sohn U. D. Hong Y. W. Choi H. C. Ha J. H. Lee K. Y. Kim W. J. . (2000). Increase of Ca(2+)i and release of arachidonic acid via activation of M2 receptor coupled to Gi and rho proteins in oesophageal muscle. Cell. Signal. 12, 215–222. doi: 10.1016/s0898-6568(99)00085-6 10781928

[B81] Sohn U. D. Park J. H. Lee T. S. Shin C. Y. Jeong J. H. Kim J. H. . (2003). Differential regulation by Ca(2+) of calmodulin- and PKC-dependent contractile pathways in cat lower oesophageal sphincter. Auton. Autacoid. Pharmacol. 23, 307–317. doi: 10.1111/j.1474-8673.2004.00302.x 15255815

[B82] Soyer T. Aktuna Z. Reşat Aydos T. Osmanoğlu G. Korkut O. Akman H. . (2010). Esophageal and gastric smooth muscle activity after carbon dioxide pneumoperitoneum. J. Surg. Res. 161, 278–281. doi: 10.1016/j.jss.2009.02.009 19524263

[B83] Soyer T. Kalkışım S. Yalcin S. Müderrisoğlu A. Taş S. T. Tanyel F. C. . (2015). The effects of acute tension increase on rat esophageal muscle contractions: An *in vitro* study. J. Pediatr. Surg. 50, 1691–1694. doi: 10.1016/j.jpedsurg.2015.01.008 25783399

[B84] Stenqvist J. Carlsson T. Winder M. Aronsson P. (2020). Functional atropine sensitive purinergic responses in the healthy rat bladder. Auton. Neurosci. 227, 102693. doi: 10.1016/j.autneu.2020.102693 32563054

[B85] Storr M. Geisler F. Neuhuber W. L. Schusdziarra V. Allescher H. D. (2000). Endomorphin-1 and -2, endogenous ligands for the mu-opioid receptor, inhibit striated and smooth muscle contraction in the rat oesophagus. Neurogastroenterol. Motil. 12, 441–448. doi: 10.1046/j.1365-2982.2000.00220.x 11012944

[B86] Storr M. Geisler F. Neuhuber W. L. Schusdziarra V. Allescher H. D. (2001). Characterization of vagal input to the rat esophageal muscle. Auton. Neurosci. 91, 1–9. doi: 10.1016/S1566-0702(01)00290-9 11515794

[B87] Strittmatter F. Gratzke C. Weinhold P. Steib C. J. Hartmann A. C. Schlenker B. . (2011). Thromboxane A2 induces contraction of human prostate smooth muscle by Rho kinase- and calmodulin-dependent mechanisms. Eur. J. Pharmacol. 650, 650–655. doi: 10.1016/j.ejphar.2010.10.052 21044618

[B88] Su P.-H. Wang T.-C. Wong Z.-R. Huang B.-M. Yang H.-Y. (2011). The expression of nestin delineates skeletal muscle differentiation in the developing rat esophagus. J. Anat. 218, 311–323. doi: 10.1111/j.1469-7580.2010.01331.x 21323914 PMC3058217

[B89] Tøttrup A. Forman A. Funch-Jensen P. Raundahl U. Andersson K. E. (1990a). Effects of transmural field stimulation in isolated muscle strips from human esophagus. Am. J. Physiol. 258, G344–G351. doi: 10.1152/ajpgi.1990.258.3.G344 2316649

[B90] Tøttrup A. Forman A. Uldbjerg N. Funch-Jensen P. Andersson K. E. (1990b). Mechanical properties of isolated human esophageal smooth muscle. Am. J. Physiol. 258, G338–G343. doi: 10.1152/ajpgi.1990.258.3.G338 2316648

[B91] Türkölmez S. Atasever T. Akmansu M. (2005). Effects of radiation therapy on oesophageal transit in patients with inner quadrant breast tumour. Nucl. Med. Commun. 26, 721–726. doi: 10.1097/01.mnm.0000171785.03403.6f 16000991

[B92] Wani S. A. Khan L. A. Basir S. F. (2018). Role of calcium channels and endothelial factors in nickel induced aortic hypercontraction in Wistar rats. J. Smooth. Muscle Res. 54, 71–82. doi: 10.1540/jsmr.54.71 30210089 PMC6135920

[B93] Wigenstam E. Forsberg E. Bucht A. Thors L. (2021). Efficacy of atropine and scopolamine on airway contractions following exposure to the nerve agent VX. Toxicol. Appl. Pharmacol. 419, 115512. doi: 10.1016/j.taap.2021.115512 33785355

[B94] Wörl J. Mayer B. Neuhuber W. L. (1994). Nitrergic innervation of the rat esophagus: focus on motor endplates. J. Auton. Nerv. Syst. 49, 227–233. doi: 10.1016/0165-1838(94)90169-4 7528756

[B95] Yao S. Gallenkamp D. Wölfel K. Lüke B. Schindler M. Scherkenbeck J. (2011). Synthesis and SERCA activities of structurally simplified cyclopiazonic acid analogues. Bioorg. Med. Chem. 19, 4669–4678. doi: 10.1016/j.bmc.2011.06.001 21719297

[B96] Zhang L. Zhao W. Zhao C. Jin H. Wang B. Wang B. (2018). Study on effects of electrical stimulation on rabbit esophageal body motility *in vivo*. Physiol. Res. 67, 275–282. doi: 10.33549/physiolres.933652 29303604

