# Supplementary material for: Characterization of calcium sources and downstream signaling pathways following electrical field stimulation of rodent longitudinal esophageal muscle
Source: Front Physiol. 2026 Aug 19;17:1907835. doi: 10.3389/fphys.2026.1907835 (PMC13533759; doi:10.3389/fphys.2026.1907835)
Supplement: Supplementary file 1 [file DataSheet1.docx]

Supplementary Material

**Suppl. Fig. 1** (A) Baseline tone in all 11 experimental groups. The total mean (2.8 mN) is shown as a gray box in the background. Asterisks denote significant differences to this mean value (t-test). (B) Percentage of the high KCl response at the end of the experiment (KCl End) to the first KCl response (KCl Start) in all 11 experimental groups. The total mean (64%) is shown as a gray box. Asterisks denote significant differences to 100 % (dotted line). N refers to the number of animals, while n denotes the number of esophageal segments used in the experiments

**Suppl. Fig. 2** Frequency-dependent contraction induced by electrical field stimulation (EFS) is shown for stimulus intensities of 30 V, 50 V, 70 V and 90 V, separately for the proximal portion (A), the middle portion (B) and the distal portion (C). Three-way ANOVA (factors stimulus intensity, frequency, location) with Holm-Sidak posthoc test detected a significant effect of location: proximal versus middle (P<0.01), proximal versus distal (P<0.05), middle versus distal (P<0.05). N refers to the number of animals, while n denotes the number of esophageal segments used in the experiments. However, the effect sizes were rather small (proximal versus middle: 0.37, proximal versus distal: 0.20, middle versus distal: 0.17).

**Suppl. Fig. 3** The addition of carbachol (c1) induces a long-lasting contraction of the esophagus, which is terminated by a flushing procedure to fresh the organ bath solution (w). The second contraction (c2) after the addition of Y-27632 (+) has a lower amplitude. Pre-incubation time with the inhibitor Y-27632 was 10 minutes.

**Suppl. Fig. 4** Comparison of EFS-induced contractions of different amplitudes and frequencies before (A) and after (B) incubation with the SERCA inhibitor CPA. A leftward shift in contractile force is observed. x-axis: Frequency (Hz), y-axis: Contractile force (mN)

**Suppl. Fig. 5** Repetitive immediately carbachol-induced (c) contractions that were terminated by refreshing the organ bath solution (w). It can be seen that the maximum contraction strength decreases over time. The second KCl response (k2) also showed a weaker contraction compared to the first KCl response (k1).

**Suppl. Table 1** Comparison between proximal, middle and distal segments of the esophagus (segment-based analysis). Data are given as mean ± SEM. Statistical comparison was performed using Kruskal-Wallis test. Abbreviations: SEM = standard error of the mean; n = number of segments; c _max_ = maximum contraction

|  | **proximal** | **middle** | **distal** | **P value** |
| --- | --- | --- | --- | --- |
| Baseline tone (mN) | 2.91 ± 0.06  (n=52) | 2.82 ± 0.05  (n=52) | 2.82 ± 0.06  (n=52) | 0.513 |
| KCl Start (mN) | 6.45 ± 0.51  (n=52) | 7.93 ± 0.65  (n=52) | 7.59 ± 0.62  (n=52) | 0.234 |
| $C_{max} reduction$ by chelerythrine (%) | 12.2 ± 4.2  (n=3) | 10.4 ± 2.4  (n=4) | 24.0 ± 0.9  (n=3) | 0.050 |
| $C_{max} reduction$ by W-7 (%) | 63.9 ± 9.7  (n=6) | 66.9 ± 5.2  (n=6) | 71.5 ± 4.5  (n=6) | 0.949 |
| $C_{max} reduction$ by chelerythrine + W-7 (%) | 56.8 ± 7.0  (n=3) | 46.9 ± 4.2  (n=3) | 44.3 ± 6.3  (n=4) | 0.791 |
| $C_{max} reduction$ by verapamil (%) | 97.0 ± 0.7  (n=6) | 93.3 ± 1.4  (n=6) | 91.0 ± 2.8  (n=6) | 0.120 |

**Suppl. Table 2** Comparison between segment-based and animal-based analysis for treatment effect and effect size. Effect size was calculated based on absolute EFS-mediated contractions.

| Treatment | Subjects | | Analysis based on segments | | Analysis based on animals | |
| --- | --- | --- | --- | --- | --- | --- |
|  | Animals  (N) | Segments  (n) | Treatment effect in 3-way ANOVA | Effect size | Treatment effect in 3-way ANOVA | Effect size |
| Time control | 4 | 18 | <0.001 | 0.30 | 0.005 | **0.56** |
| DMSO | 10 | 20 | <0.001 | 0.10 | <0.001 | **0.13** |
| Atropin | 3 | 18 | <0.001 | 0.15 | <0.001 | **0.14** |
| Y27632 | 4 | 18 | <0.001 | 0.22 | 0.079 | **0.36** |
| 2-APB | 4 | 8 | <0.001 | -0.51 | <0.001 | **-0.56** |
| DHBP | 4 | 8 | 0.004 | 0.23 | 0.074 | **0.28** |
| 2-APB + DHBP | 3 | 10 | 0.042 | -0.03 | <0.001 | **-0.17** |
| Chel | 3 | 10 | <0.001 | 0.70 | <0.001 | **5.43** |
| W-7 | 3 | 18 | <0.001 | 1.34 | <0.001 | **2.50** |
| Chel + W7 | 3 | 10 | <0.001 | 1.96 | <0.001 | **1.76** |
| Chel vs. Chel + W-7 | 3* | 10* | <0.001 | 1.16 | <0.001 | **1.50** |
| W-7 vs. Chel + W-7 | 3* | 18/10* | <0.05 | -1.20 | <0.001 | **-1.56** |
| Verapamil | 4 | 18 | <0.001 | 2.89 | <0.001 | **4.26** |
